# Upper tract urothelial carcinoma has a luminal-papillary T-cell depleted contexture and activated FGFR3 signaling

**DOI:** 10.1038/s41467-019-10873-y

**Published:** 2019-07-05

**Authors:** Brian D. Robinson, Panagiotis J. Vlachostergios, Bhavneet Bhinder, Weisi Liu, Kailyn Li, Tyler J. Moss, Rohan Bareja, Kyung Park, Peyman Tavassoli, Joanna Cyrta, Scott T. Tagawa, David M. Nanus, Himisha Beltran, Ana M. Molina, Francesca Khani, Juan Miguel Mosquera, Evanguelos Xylinas, Shahrokh F. Shariat, Douglas S. Scherr, Mark A. Rubin, Seth P. Lerner, Surena F. Matin, Olivier Elemento, Bishoy M. Faltas

**Affiliations:** 1 Department of Pathology and Laboratory Medicine, Weill Cornell Medicine, New York, NY 10065 USA; 2 Department of Urology, Weill Cornell Medicine, New York, NY 10065 USA; 3 Englander Institute for Precision Medicine, Weill Cornell Medicine, New York, NY 10021 USA; 4 Department of Medicine, Division of Hematology and Medical Oncology, Weill Cornell Medicine, New York, NY 10065 USA; 5 Department of Physiology and Biophysics, Weill Cornell Medicine, New York, NY 10065 USA; 6 Institute for Computational Biomedicine, Weill Cornell Medicine, New York, NY 10021 USA; 7 Department of Bioinformatics and Computational Biology, The University of Texas MD Anderson Cancer Center, Houston, TX 77030 USA; 8 Sandra and Edward Meyer Cancer Center at Weill Cornell Medicine, New York, NY 10065 USA; 9 Department of Urology, Cochin Hospital, APHP, Paris Descartes University, Paris, 75014 France; 10 Department of Urology, Medical University of Vienna, Vienna, 1190 Austria; 11 Department of Biomedical Research, University of Bern, Bern, 3008 Switzerland; 12 Scott Department of Urology, Baylor College of Medicine, Houston, TX 77030 USA; 13 Department of Urology, The University of Texas MD Anderson Cancer Center, Houston, TX 77030 USA; 14 Department of Cell and Developmental Biology, Weill Cornell Medicine, New York, NY 10065 USA

**Keywords:** Cancer genomics, Molecular medicine, Bladder cancer

## Abstract

Upper tract urothelial carcinoma (UTUC) is characterized by a distinctly aggressive clinical phenotype. To define the biological features driving this phenotype, we performed an integrated analysis of whole-exome and RNA sequencing of UTUC. Here we report several key insights from our molecular dissection of this disease: 1) Most UTUCs are luminal-papillary; 2) UTUC has a T-cell depleted immune contexture; 3) High FGFR3 expression is enriched in UTUC and correlates with its T-cell depleted immune microenvironment; 4) Sporadic UTUC is characterized by a lower total mutational burden than urothelial carcinoma of the bladder. Our findings lay the foundation for a deeper understanding of UTUC biology and provide a rationale for the development of UTUC-specific treatment strategies.

## Abstract (web summary)

Upper tract urothelial carcinoma (UTUC) is an aggressive cancer and largely uncharacterised cancer. Here, Faltas and colleagues report its distinctive molecular and immune landscape compared to urothelial carcinoma of the bladder and explore the role of FGFR3 signaling in UTUC biology.

## Introduction

Upper tract urothelial carcinoma (UTUC) accounts for 5–10% of all urothelial carcinomas (UCs)^1^. UTUC is a distinct clinical entity with an aggressive clinical behavior and a more advanced presentation compared to urothelial carcinoma of the bladder (UCB)^1^. Recently, the Cancer Genome Atlas (TCGA) study classified UCB into five molecular subtypes (luminal-papillary, luminal-infiltrated, luminal, basal/squamous, neuronal). Since TCGA did not include UTUC^2^, it is currently unknown whether UTUC recapitulates the same molecular subtypes^2–6^. Furthermore, our understanding of the immune milieu of UTUC is incomplete.

These knowledge gaps have hindered the development of effective UTUC-specific therapeutic strategies. To dissect the central biological features of UTUC’s tumor and immune cell compartments, we analyzed whole-exome sequencing (WES) and RNA sequencing (RNAseq) data from high-grade UTUC tumors from patients at three different institutions [Weill Cornell Medicine (WCM), Baylor College of Medicine and MD Anderson Cancer Center (BCM–MDACC)]. We used whole-exome and RNAseq data from UCB tumors from the TCGA cohort as a comparison cohort^5^. This enabled us to define the biological differences between UC arising from the upper and lower urinary tracts and to gain insights into the unique mechanisms that drive UTUC biology. We show that UTUC is predominantly luminal-papillary and T-cell depleted. We identify FGFR3 as a putative regulator of UTUC’s immune contexture through attenuation of interferon gamma (IFNG) signaling. Finally, we report that the tumor mutational burden in sporadic UTUC is lower than UCB, despite reduced expression of DNA mismatch repair (MMR) transcripts and proteins.

## Results

### UTUC and UCB mutational profiles

**Fig. 1:**
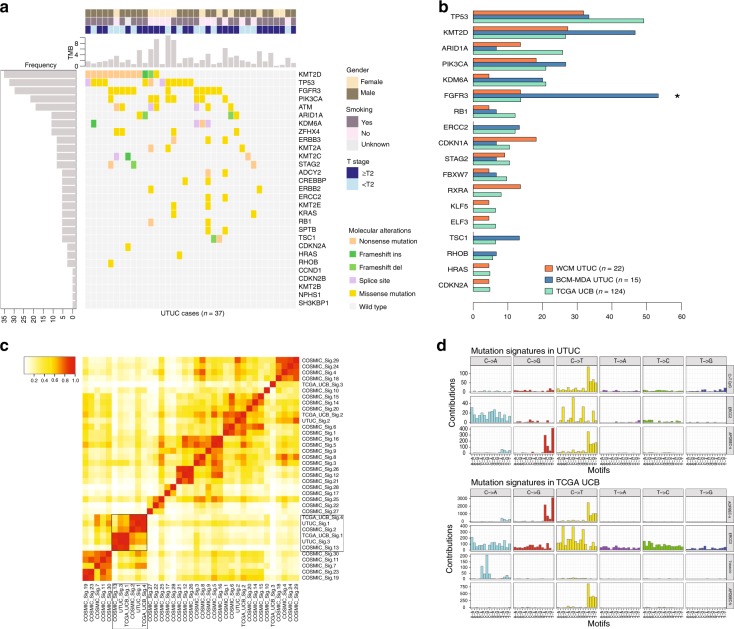
Genomic differences between upper tract urothelial carcinoma (UTUC) and urothelial bladder carcinoma (UCB). **a** Prevalence of frequent somatic genomic alterations in 37 patients with UTUC from the Weill Cornell Medicine (WCM UTUC) and Baylor College of Medicine–MD Anderson Cancer Center (BCM–MDACC UTUC) cohorts. The somatic mutational rate for each tumor is represented by vertical barplots (total number of non-silent mutations per megabase (MB)). Patient and tumor characteristics are represented on the top right. **b** Horizontal barplot showing differences in mutational frequencies between WCM UTUC (orange bars), BCM-MDA UTUC (blue bars), and TCGA UCB (green bars) cohorts for frequently mutated genes. Asterisk indicates statistically significant changes in paired comparison between BCM-MDA UTUC and TCGA UCB (*Fisher’s Exact test *P* = 0.001). **c** Heatmap of a cosine similarity matrix of COSMIC mutational signatures and observed mutational signatures in UTUC (WCM, BCM-MDA) and TCGA UCB cohorts. UTUC signature clusters with COSMIC signatures 2, 13 (APOBEC-associated). **d** Dominant mutational signatures in UTUC (top panel) include the C>T CpG signature, attributable to mutagenesis via defective mismatch repair (MMR) (COSMIC signature 6), the ERCC2 signature, consistent with COSMIC signature 5, and the APOBEC signatures (COSMIC signatures 2 and 13) resulting from the endogenous cytidine deamination induced by the APOBEC3 enzyme family. TCGA UCB mutational signatures are shown for comparison (bottom panel)

We performed whole exome sequencing (WES) of 37 UTUC primary tumor–normal pairs. We only included patients with high-grade UTUC tumors. Most patients were former or current smokers (64.8%) (Supplementary Table 1). We identified *FGFR3* mutations in 11/37 (29.7%) (Fig. 1a), a significantly higher frequency compared to the 17/124 (13.7%) mutations detected in TCGA UCB (Wilcoxon test *P* = 0.04) (Fig. 1b). In contrast, we found no significant difference in the prevalence of mutations in chromatin modifying (*KMT2D, ARID1A, KDM6A*), receptor tyrosine kinase pathway (*PIK3CA, HRAS*), transcription factor (RXRA, KLF5, ELF3), and cell cycle regulation (*TP53*, *RB1*, *CDKN1A*, *CDKN2A*) genes between our UTUC and TCGA UCB cohorts (Fig. 1a, b).

To define the mutagenic mechanisms that shape the genomic landscape of UTUC, we performed a mutational signature analysis to identify the prevalent Catalog of Somatic Mutations in Cancer (COSMIC) signatures in the WCM UTUC, BCM-MDA UTUC, and TCGA UCB cohorts^7^. We identified the COSMIC APOBEC-associated signatures 2 and 13 as the dominant mutational signatures in UTUC (Fig. 1c). We also identified C>T transitions at CpG dinucleotides. This signature is characterized by high numbers of small indels at mono/polynucleotide repeats and is associated with defective MMR (Fig. 1d). Another mutational signature in UTUC was found to be related to defective nucleotide excision repair (NER)^8^ (Fig. 1d). Collectively, these data suggest that these three mutational processes (APOBEC, MMR, NER) (Fig. 1d) are responsible for the majority of mutations in UTUC.

### Somatic downregulation of DNA damage repair (DDR) genes in UTUC

**Fig. 2:**
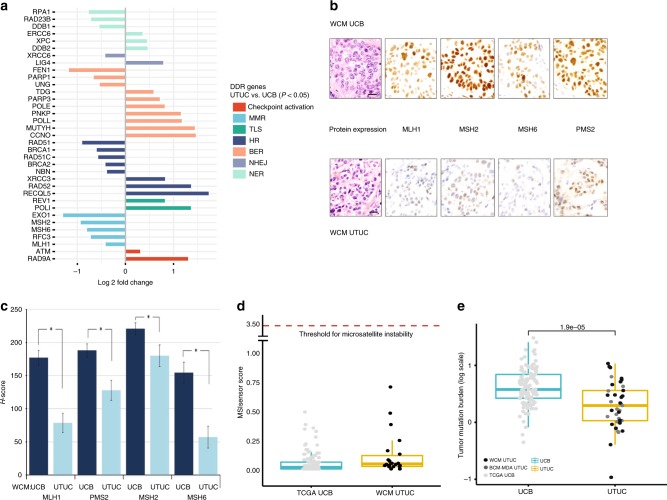
UTUC is characterized by decreased expression of canonical MMR proteins. **a** Comparative histogram of DNA damage response (DDR) genes with statistically significant differential expression (log_2_ fold) highlighting significantly lower expression of mismatch repair (MMR) pathway genes (*MLH1, MSH2, MSH6, RFC3, EXO1*) in WCM UTUC compared to TCGA UCB (one-way ANOVA log2FC adjusted *P* ≤ 0.05). **b** Representative micrographs (magnification ×400) showing lower expression of MMR proteins in a UTUC tumor compared to a UCB tumor by immunohistochemistry. Scale bars represent 25 µM. **c** Mean *H*-scores of MMR proteins are significantly lower in WCM UTUC tumors compared to WCM UCB tumors. Error bars show standard deviation (S.D.) for each MMR protein. Asterisks indicate statistically significant changes in paired comparison (*t*-test *P* < 0.05). **d** Mean microsatellite instability (MSI) score is below the threshold of 3.5 in both WCM UTUC and TCGA UCB tumors. **e** Mean total mutational burden (TMB), expressed in log scale is lower in UTUC (WCM, BCM-MDA) compared to TCGA UCB tumors (Mann–Whitney test *P* = 1.9 × 10^−5^). The horizontal lines within the boxes in the boxplots indicate the mean, boundaries of the boxes indicate the 25th-percentile and 75th-percentile, and the whiskers indicate the highest and lowest values of the results

The association between germline mutations in MMR genes that cause microsatellite instability (MSI) and Lynch syndrome and increased susceptibility to the development of UTUC is well established^9–11^. However, it is unclear whether non-Lynch (sporadic) UTUC patients have defective DDR and an increased mutational burden. To define whether somatic dysregulation of DDR genes could play a similar role in inducing a hypermutated phenotype in non-Lynch UTUC patients, we assessed the mRNA expression level of DDR pathway genes in UTUC (WCM, BCM-MDA) and UCB (TCGA). We focused our analysis on identifying differentially expressed genes in the canonical DDR pathways [MMR, base-excision repair (BER), NER, homologous recombination (HR), non-homologous end joining (NHEJ), Fanconi anemia (FA), translesion synthesis (TLS)] comparing UTUC with UCB (Fig. 2a). We identified a significant somatic dysregulation of 35 canonical DDR genes in UTUC (Fig. 2a). We analyzed germline WES data from patients in our UTUC cohorts (WCM, BCA-MDA) and identified no germline mutations in the canonical MMR genes (*MLH1, PMS2, MSH2, MSH6*). Interestingly, we observed significantly lower somatic mRNA expression of three canonical MMR genes: *MLH1*, *MSH2*, and *MSH6* in UTUC tumors compared to TCGA UCB tumors (Fig. 2a). To determine whether this decreased mRNA levels further translated into a low expression of MMR proteins, we used immunohistochemistry (IHC) to quantify their expression in WCM UTUC tumors (*n* = 16) compared to stage-matched WCM UCB tumors (*n* = 14). We found that the levels of MLH1, PMS2, MSH2, and MSH6 proteins were significantly lower in UTUC tumors (Fig. 2b, c). This confirmed that lower expression of these proteins is a characteristic feature of UTUC even in the absence of germline or somatic mutations in the respective genes in the WCM UTUC cohort.

To determine whether downregulation of these proteins impaired DNA MMR, we examined WES data for short tandem repeats (MSI) using the MSI sensor program^12^ that calculates the percentage of unstable microsatellites from UTUC and UCB tumor–normal paired WES data. Surprisingly, the median MSI sensor scores were similar in WCM UTUC and TCGA UCB. Both were below the previously defined threshold of 3.5, which was shown to accurately classify microsatellite unstable tumors^12^ (Fig. 2d). Furthermore, WCM UTUC samples harbored a significantly lower mean total mutational burden (TMB) compared to TCGA UCB tumors (2.91 versus 5.46 mutations per MB) (Fig. 2e). This suggests that the somatic downregulation of MMR proteins is insufficient to produce MSI and is not a major driver of mutagenesis in non-Lynch UTUC. Collectively, these findings indicate that the decrease in the mRNA and protein levels of MMR genes in sporadic UTUC does not translate into MSI or a higher TMB.

### UTUCs are predominantly luminal-papillary

**Fig. 3:**
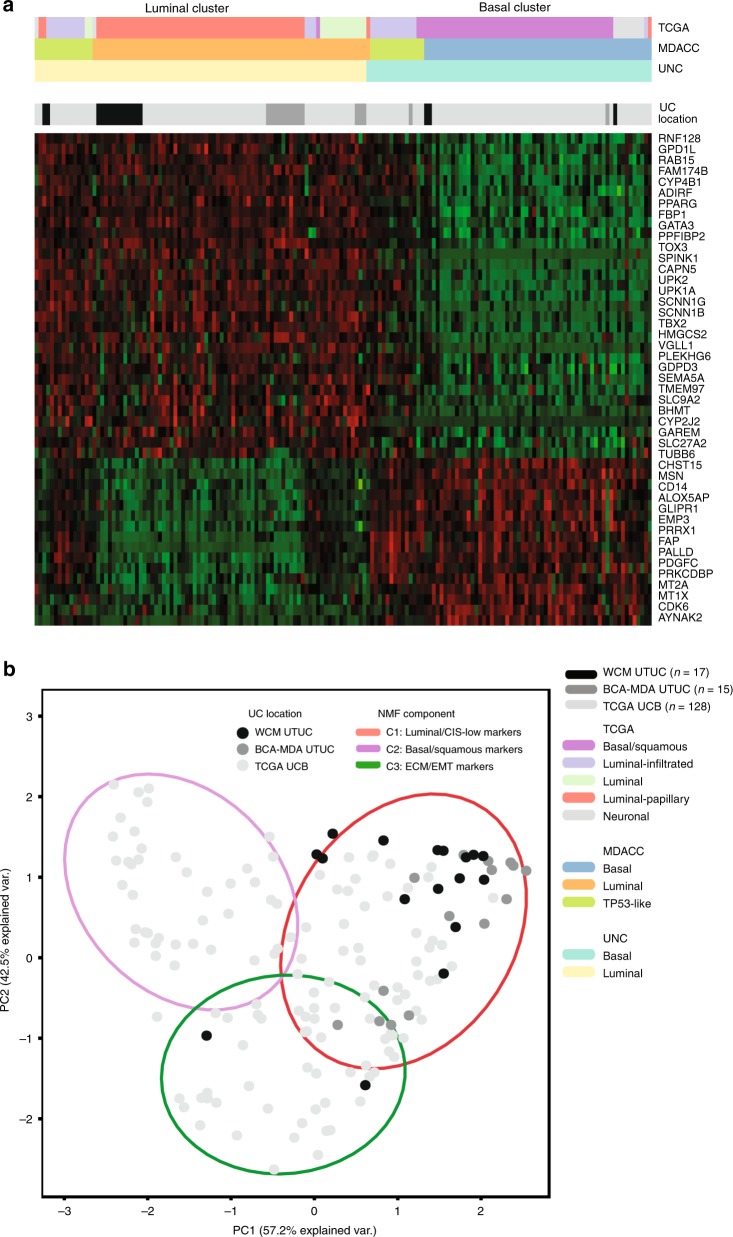
UTUC is predominantly luminal. **a** Supervised consensus clustering and heatmap of mRNA expression data from WCM UTUC, BCM-MDA UTUC, and TCGA UCB metadataset. BASE47 classifier (UNC) genes are listed on the right. Assigned TCGA, MDACC, and UNC clusters are represented on top (color key, bottom right). WCM UTUC and BCM-MDA UTUC cluster with the luminal subtype (yellow vertical bars) by UNC criteria, luminal subtype by MDACC criteria (orange horizontal bars) and luminal-papillary subtype by TCGA classification (red horizontal bars). **b** Non-negative matrix factorization (NMF) of WCM UTUC, BCM-MDA UTUC, and TCGA UCB tumors segregates gene expression along three principal components: basal/squamous (purple), luminal/CIS-low (pink), and extracellular matrix/epithelial–mesenchymal transition (ECM/EMT) (green). WCM UTUC tumors represented as black dots and BCM-MDA tumors represented as dark gray dots cluster with the luminal/CIS-low component

To  characterize the gene expression profiles which define UTUC, we generated an RNAseq meta-dataset from 32 UTUC tumors and the TCGA UCB cohort. To ensure homogeneity, we performed sample normalization of the data prior to standardization and fitted the quantiles of each sample’s raw data to be similar. We also compared the *z*-scores of the mRNA expression of 40 housekeeping genes among tumor samples from WCM UTUC, BCM-MDA UTUC, and TCGA UCB and found no significant differences  for the majority of these genes (Supplementary Fig. 1). Using the University of North Carolina (UNC) 47-gene signature (BASE47) classifier^3^, we found that 27/32 (84.3%) of the UTUC tumors clustered with the luminal subtype (Fig. 3a and Supplementary Table 2) as opposed to only 59/128 (46.1%) in UCB^2^. To ensure that this luminal expression pattern is a consistent biological property of UTUC, we confirmed this finding using several different methods. First, we interrogated the same meta-dataset using two additional validated classifiers of urothelial carcinoma subtypes. When we applied the MDACC classifier which divides UC into luminal, basal, and p53-like subtypes^4^, we found that 22/32 (68.7%) of UTUC tumors clustered with the luminal subtype (Supplementary Fig. 2 and Supplementary Table 2) versus only 47/128 (36.7%) of UCB^2^. Using the recent TCGA classifier which segregates UC into luminal-papillary, luminal-infiltrated, luminal, basal-squamous, and neuronal subtypes^2^, we confirmed that UTUC has a luminal-papillary phenotype (20/32, 62.5%), with the majority of remaining UTUC tumors also exhibiting a luminal expression profile (8/12, 67%) (Supplementary Fig. 3 and Supplementary Table 2). This is in contrast to 35/128 (27.3%) of luminal-papillary UCB tumors in the TCGA UCB cohort, with only a minority of the rest (35/93, 37.6%) also segregating with luminal or luminal-infiltrated subtypes^2^. To confirm these findings using a different approach, we used non-negative matrix factorization (NMF)^13^, a sensitive unsupervised statistical method to dissect and extract the key biological features of UTUC from our high dimensional RNAseq dataset^13^. The NMF analysis revealed three principal components characterized by luminal/carcinoma in situ (CIS)-low, basal/squamous-like, and extracellular matrix (ECM)/epithelial–mesenchymal transition (EMT)-related gene sets^2^. Principal component analysis (PCA) was then performed on the coefficients obtained from NMF to visualize the separation of the three components (Fig. 3b). This demonstrated that the luminal-papillary component is a defining feature of UTUC. Our results suggest that the majority of UTUCs represent a distinct subset within the continuum of UC differentiation that shares similar characteristics with the luminal-papillary subtype of UCB.

### UTUC has a T-cell depleted immune contexture

**Fig. 4:**
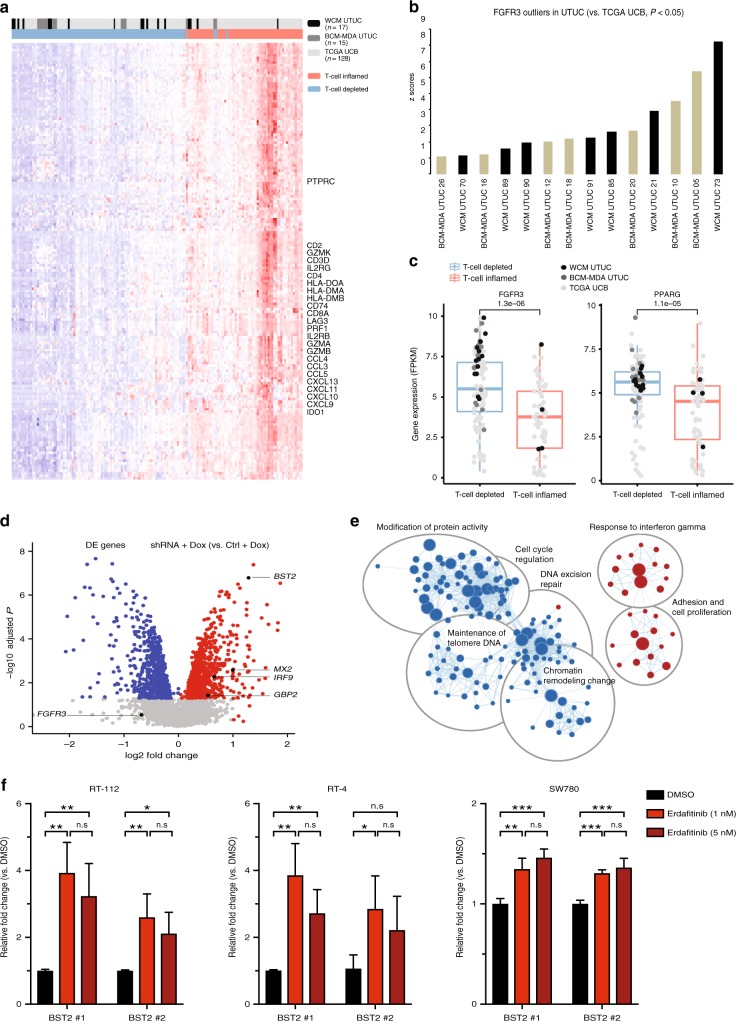
FGFR3 plays an important role in the T-cell-depleted immune contexture of UTUC. **a** UTUC is T-cell depleted. Supervised consensus clustering of WCM UTUC, BCM-MDA UTUC, and TCGA UCB tumors according to a 170-immune gene signature classifies tumors into T-cell depleted (with lower expression of classifier genes), and T-cell inflamed (with higher expression of classifier genes) clusters (Fisher’s exact test *P* = 9 × 10^-5^). **b** FGFR3 is an expression outlier in UTUC tumors. WCM UTUC and BCM-MDA UTUC tumors are represented on the *x*-axis, and normalized *z*-scores of gene’s expression represented on the *y*-axis. **c** Boxplots of mean expression of *FGFR3* and *PPARG* genes [in Fragments Per Kilobase of transcript per Million mapped reads (FPKM)] within the T-cell-depleted versus T-cell inflamed clusters (*FGFR3*: Wilcoxon test *P* = 1.3 × 10^−6^; *PPARG*: Wilcoxon test *P* = 1.1 × 10^−5^). The horizontal lines within the boxplots indicate the mean, boundaries of the boxes indicate the 25th-percentile and 75th-percentile, and the whiskers indicate the highest and lowest values of the results. **d** Interferon gamma (IFNG)-response genes are upregulated in response to FGFR3 knockdown. Volcano plot of differential fold expression of genes (logFC < 0 vs. logFC > 0; *t*-test adjusted *P* < 0.05) in *FGFR3* shRNA + Doxycycline compared to control + Doxycycline UCB RT-112 cells (*BST2: P* < 0.001; *GBP2: P* = 0.038; *IRF9*: *P* = 0.005; *MX2*: *P* = 0.003). **e** Enrichment map of cancer-related pathways with significant positive and negative enrichment in *FGFR3* shRNA UCB RT-112 cells. Node size corresponds to the number of genes within each gene set. The up-regulated nodes were represented in red while the down-regulated clusters were represented in blue. Overall, 476/3534 gene sets were upregulated, and 651/3534 gene sets were downregulated (*t*-test *P* < 0.05; false discovery rate (FDR) < 0.25). Several IFNG response gene sets were enriched after *FGFR3* blockade (*t*-test *P* < 0.001; FDR = 0.062). **f** Pharmacologic inhibition of FGFR3 upregulates IFNG-response gene *BST2* using two different primer pairs (BST2 #1, BST2 #2). Barplots showing relative fold increase (mean ± SD) of mRNA levels of *BST2* (BST2 #1 and BST2 #2) after treatment with erdafitinib at 1 and 5 nM compared to DMSO vehicle in RT-112, RT-4, and SW780 cells. No statistically significant differences were observed in the expression of *BST2* between the 1 and 5 nM erdafitinib conditions in any of the tested cell lines (statistical significance level is denoted by asterisks *t-test *P* < 0.05, **P < 0.01, ***P < 0.001, n.s: non-significant). Error bars show standard deviation (S.D.) for each condition

The immune contexture of tumors is an essential determinant of the host’s anti-cancerimmune response^14^ and clinical outcomes. To dissect the gene expression profile of UTUC that characterizes its immune contexture, we developed a 170-gene classifier comprising key immune genes (Supplementary Table 3). This classifier separated tumors independent of their anatomical origin into T-cell inflamed, and T-cell-depleted clusters. Interestingly, the majority of UTUC (WCM, BCM-MDA) tumors (28/32, 87.5%) were T-cell depleted (Fig. 4a) with consistent downregulation of T-cell related (*CD8A, CCL2, CCL3, CCL4, CXCL9, CXCL10*)^15^ and *IFNG* signaling genes^16^ (Fig. 4a). In contrast, TCGA UCB tumors were almost evenly distributed between T-cell inflamed (57/128, 44.5%) and T-cell depleted (71/128, 55.5%) immune subtypes (UTUC vs. UCB (Fisher’s exact test *P* = 0.0009) (Fig. 4a).

### FGFR3 is a putative driver of UTUC’s immune-depleted contexture

To identify the signaling pathways that characterize UTUC’s immune contexture, we performed gene expression analysis to detect outliers. We detected outlier *FGFR3* mRNA expression in 14/32 (43.7%) of the tumors in our UTUC (WCM, BCM-MDA) cohorts (Fig. 4b). We identified nine activating missense mutations in these tumors. These findings suggest that activated FGFR3 signaling potentially plays a prominent role in the distinct gene expression profile of UTUC.

We then went on to assess whether *FGFR3* is differentially expressed between T-cell inflamed and T-cell-depleted tumors. Indeed, we identified significantly higher *FGFR3* expression in the T-cell-depleted cluster which harbored the majority of the UTUC tumors (Fig. 4c). We interrogated the functional link between FGFR3 signaling and the T-cell-depleted phenotype observed in UTUC. We reanalyzed a previously published dataset of mRNA expression from the RT-112 UC cell line following doxycycline (dox)-inducible short hairpin RNA (shRNA) knockdown of *FGFR3*^17,18^. We found that several IFNG response genes including *BST2, MX2, IRF9, GBP2* were upregulated after *FGFR3* knockdown (Fig. 4d). Confirming these results, we found that *BST2* and *IRF9* were significantly downregulated in the T-cell-depleted cluster which harbored the majority of UTUCs in our patient cohort (Supplementary Fig. 4). We also found a statistically significant upregulation of other IFNG response genes in the *FGFR3* shRNA + dox dataset when compared to the control (ctl) + dox dataset (Fig. 4e). To confirm this observation and define whether pharmacologic inhibition of FGFR3 will have a similar effect on IFNG response genes (Supplementary Table 4), we tested the effects of erdafitinib, a small molecule FGFR3 inhibitor, in three different UC cell lines (RT-112, RT-4, and SW780). These cell lines harbor FGFR3 fusions (RT-112, RT-4: FGFR3-TACC3; SW780: FGFR3-BAIAP2L1) resulting in constitutively activated FGFR3 signaling^19^. We found that treatment with erdafitinib led to a significant upregulation of *BST2*, a hallmark of activated interferon signaling (Fig. 4f). Collectively, these findings show that FGFR3 plays an important role in shaping the T-cell-depleted phenotype in UTUC in a cancer-cell autonomous manner.

## Discussion

We performed a comprehensive genomic and transcriptomic analysis of UTUC to identify the key biological features that differentiate UTUC from UCB. We found that the majority of UTUC tumors are luminal-papillary and T-cell depleted.

Previous studies identified a link between Lynch syndrome caused by germline mutations in MMR genes and UTUC^9–11^. However, the vast majority of UTUCs arise sporadically (in non-Lynch syndrome patients). Our analysis aimed to define whether sporadic UTUC patients had MSI or a higher TMB, which are independent predictors of response to immune checkpoint inhibition^20,21^. Our study revealed a lower TMB in non-Lynch syndrome UTUC compared to UCB. We found that the lower expression of canonical MMR mRNA and proteins in non-Lynch UTUC was insufficient to produce significant MSI or a higher TMB compared to UCB. Unlike the complete absence of MSH2, MLH1, MSH6, or PMS2 or MLH1 protein expression observed in Lynch syndrome patients caused by loss-of-function germline mutations, the incomplete loss of these proteins observed in sporadic UTUC is not sufficient to cause MSI^22^. Even low MMR protein expression is adequate for maintaining functional MMR and preserving microsatellite stability^23^. These results are also consistent with a recent study showing MSI sensor scores < 3.5 in the majority of non-Lynch syndrome UTUC patients^24^. Taken together, our findings suggest that, contrary to the prevalent notion, sporadic UTUC is not hypermutated. This is especially important when considered in conjunction with our data showing that the majority of sporadic UTUCs are also consistently luminal-papillary and T-cell depleted. This new understanding of the mutational landscape and immune contexture in non-Lynch syndrome UTUCs (which constitute the majority of cases) further explains the lack of higher response rates of UTUC compared to UCB in clinical trials of immune checkpoint inhibitors (ICIs)^20,21^. In a prospective phase 2 study of advanced, post-platinum UC patients (including UTUC), atezolizumab demonstrated the lowest response rates in the luminal-papillary (cluster I) subtype compared to other subtypes^20^. In a different trial, response to nivolumab was lower in the luminal 1 (cluster I) UC tumors with low expression of IFNG signature genes^15^.

We identified a putative role for upregulated FGFR3 in UTUC in shaping the immune contexture of T-cell-depleted UTUC tumors. This observation is consistent with the previously described enrichment of the *FGFR3* gene signature in luminal UCB tumors^2–6,25^ and the association of FGFR upregulation with T-cell depletion in tumors of pancreatic and breast origin^14^. We observed a consistent increase in *BST2* following pharmacologic FGFR3 inhibition in three different UC cell lines that harbor activating FGFR3 fusions. BST2 is a viral restriction factor which is canonically induced by interferon^26^.This is also consistent with the role of FGFR3 in blocking the Y701 tyrosine phosphorylation required for STAT1 activation^27^. Taken together, these findings provide putative mechanistic links between FGFR3 and IFNG signaling and suggest that FGFR3 inhibition potentially remodels the immune contexture of UTUC by upregulating interferon response genes to reverse its T-cell-depleted phenotype.

Our observations also provide a rationale for combining FGFR3 inhibitors with PD-1/PD-L1 inhibitors as a targeted therapeutic strategy to modulate the T-cell-depleted phenotype of UTUC. Preliminary clinical trial results using two pan-FGFR inhibitors, erdafitinib, and BGJ398 in several cancers enriched for *FGFR* genomic alterations including urothelial carcinoma, are encouraging^28,29^. Erdafitinib was granted  accelerated approval by the FDA in relapsed/refractory metastatic bladder cancer on the basis of phase 2 trial results showing a response rate of 32.2% in 87 patients with tumors that harbored actionable *FGFR* alterations^30^. Our findings suggest that clinical trials of FGFR3 inhibitors as single agents or in combination with immune checkpoint blockade as a UTUC-targeted therapeutic strategy is warranted. This strategy is also potentially applicable to other tumor types harboring *FGFR3*-activating molecular alterations.

Our study has multiple strengths. We used different approaches to confirm that the predominantly luminal-papillary phenotype of UTUC is a consistent biological feature. In a previous study, unsupervised clustering of RNAseq data from both high-grade and low-grade UTUC was used to divide UTUC into four molecular subtypes^31^. Here, we used an alternative approach to position high-grade UTUC within the continuum of UC biology. Our study also has several limitations. Even though we included patients from three major academic institutions, our cohort is still limited by sample size due to the relative rarity of this tumor type. Further confirmation of our findings in larger UTUC cohorts is warranted. Future studies also need to examine the stability of the molecular profiles we identified across matched primary and metastatic UTUC tumors.

In summary, our findings lay the foundation for a deeper understanding of the key features of the biology of UTUC. Based on this knowledge, we provide a roadmap for the rational clinical development of targeted and immunotherapeutic strategies that are specific to UTUC but also potentially applicable to other tumor types harboring *FGFR3*-activating molecular alterations.

## Methods

### Patient enrollment and tissue acquisition

The study was approved by our Institutional Review Boards (Weill Cornell Medicine (WCM)/New York-Presbyterian (NYP) IRB protocols for Tumor Biobanking—0201005295, GU tumor Biobanking—1008011210, Urothelial Cancer Sequencing—1011011386, Comprehensive Cancer Characterization by Genomic and Transcriptomic Profiling—1007011157 and Precision Medicine—1305013903). Banked excess tissue was collected from nephroureterectomy specimens of patients with a diagnosis of high-grade UTUC. UTUC high-grade samples were obtained from patients under protocols approved by institutional review boards using endoscopic biopsy or surgical resection at BCM and MDACC^31^. All tumor samples consisted of conventional UC. Samples were selected based on pathologic diagnosis according to standard guidelines for UTUC^1,32^. All pathology specimens were reviewed and reported by board-certified genitourinary pathologists in the Department of Pathology at WCM/NYP, BCM and MDA. Clinical charts were reviewed by the authors (P.J.V., T.J.M., S.F.M., S.P.L., B.M.F.) to record patient demographics, tobacco use, treatment history, anatomic site, the presence of concurrent bladder cancer, pathologic grade and stage using tumor, node, metastasis (TNM) system. DNA for WES was extracted from tumors and matched normal tissues and RNA was purified from tumors for RNAseq.

### DNA extraction and WES

For WCM UTUC samples, we used our established WES protocol^33,34^. After macrodissection of target lesions, tumor DNA was extracted from formalin-fixed, paraffin-embedded (FFPE) or cored OCT-cryopreserved tumors using the Promega Maxwell 16 MDx (Promega, Madison, WI, USA). Germline DNA was extracted from normal kidney tissue adjacent to the tumor, using the same method. Pathological review by one of the study pathologists (B.D.R., J.M.M., M.A.R.) confirmed the diagnosis and determined tumor content. A minimum of 200 ng of DNA was used for WES. DNA quality was determined by TapeStation Instrument (Agilent Technologies, Santa Clara, CA) and was confirmed by real-time PCR before sequencing. Sequencing was performed using Illumina HiSeq 2500 (2 × 100 bp). A total of 21,522 genes were analyzed with an average coverage of 85× using Agilent HaloPlex Exome (Agilent Technologies, Santa Clara, CA). For BCM-MDA samples, DNA was purified from tumor and matched normal tissues and used for WES^31^.

### WES data processing pipeline

All the WCM samples data were processed through the computational analysis pipeline of the Institute for Precision Medicine at Weill Cornell, New York Presbyterian Hospital (IPM-Exome-pipeline)^32^. Raw reads quality was assessed with FASTQC^33^. Pipeline output includes segment DNA copy number data, somatic copy-number aberrations (CNAs) and putative somatic single-nucleotide variants (SNVs). Bioinformatic analysis of BCM-MDA samples data was performed^31^.

### Single nucleotide variants

We developed a consensus somatic SNVs calling pipeline to enhance the accuracy of these calls for WCM samples. SNVs were identified in the paired tumor–normal samples using MuTect2, Strelka, VarScan, and SomaticSniper, and only the SNVs identified by at least two mutation callers were retained. Indels (insertions or deletions) were identified using Strelka and VarScan and only those identified by both tools were retained. The identified somatic alterations were further filtered using the following criteria: (a) read depth for both tumor and matched normal samples was ≥ 10 reads, (b) the variant allele frequency (VAF) in tumor samples was ≥ 5% and >3 reads harboring the mutated allele, (c) the VAF of matched normal was ≤ 1% or there was just one read with mutated allele. The variants were annotated using Oncotator (version 1.9); the dbSNPs amongst the mutation calls, unless also found in the COSMIC database, were filtered out. For the IPMs samples, the promiscuous mutation calls, previously identified internally as artifacts for Haloplex were also excluded from the final list of mutations. Tumor mutation burden (TMB) was calculated for each sample as the number of mutations divided by the number of bases in the coverage space per million. Somatic mutations were called via a standard cancer analysis pipeline at the BCM Human Genome Sequencing Center and by using VARSCAN2 for BCM-MDA samples^31^.

### Mutational signature analysis

Somatic alterations identified from the WES analysis pipeline were used to identify underlying patterns of mutational signatures. The nonsynonymous SNVs were classified into the six base substitution classes and the bases immediately to the 5′ and 3′ of the mutated base gave 16 different mutational contexts; hence there were 96 different base substitution patterns in total. All base substitutions were reported in context of pyrimidines and in 5′ to 3′ direction. The signatures were identified using the counts of 96 base substitutions for each sample, based on the Bayesian NMF^8^. The signatures discovered in the WCM UTUC, BCM-MDA UTUC and TCGA UCB cohorts were compared to the 30 COSMIC signatures using hierarchal clustering of cosine similarity amongst these signatures with ‘ward.D2’ linkage.

### MMR histochemical expression and *H*-score calculation

Expression of MMR proteins was assessed in 16 WCM UTUC tumors and 14 matched archival WCM UCB. IHC was performed on 4-μm-thick formalin-fixed paraffin-embedded tissue sections using a Leica Bond III automated stainer. Mouse antibodies against MLH1 (G168-728, 1:25 dilution, BD Biosciences), PMS2 (A16-4, 1:100 dilution, BD Biosciences), MSH2 (FE11, 1:200, EMD Millipore), and MSH6 (44/MSH6, 1:200, BD Biosciences) were used. IHC slides were scanned at ×200 total magnification using a single z-plane via an Aperio AT2 whole slide scanner (Leica Biosystems, San Diego, CA, USA). The scanned images were loaded onto the HALO^TM^ imaging analysis platform (Indica Labs, Corrales, New Mexico, USA). Study pathologists manually selected tumor areas for automated image scoring, and the HALO^TM^ analysis software determined the staining intensity of each tumor cell (0, 1+, 2+, 3+) and percentage of tumor cells for each intensity level. *H*-scores were then calculated using the formula [1×(% of cells with intensity of 1+)+2×(% cells 2+)+3×(% cells 3+)] with possible scores thus ranging from 0 to 300.

### Computational detection of MSI

MSI in WCM UTUC and TCGA UCB samples was detected by MSI sensor. MSI sensor is a software tool that quantifies MSI in paired tumor–normal genome sequencing data and reports the somatic status of corresponding microsatellite sites in the human genome^12^. MSIsensor score was calculated by dividing the number of microsatellite unstable by the total number of microsatellite stable (MS) sites detected. The cut-off for defining MSI-high (MSI-H) versus MS stable (MSS) samples was 3.5 (MSI-H > 3.5, MSS < 3.5)^12^.

### Germline variant calling pipeline

Germline samples used in this study were normal tissue from fresh frozen or formalin-fixed paraffin-embedded tissue from nephroureterectomy archival specimens of patients with a diagnosis of UTUC at WCM^35^. We applied a germline variant calling pipeline based on the Burrows–Wheeler Aligner (BWA), and the Genome Analysis Toolkit (GATK), for base recalibration, realignment around indels and variant calling. We devised a variant filtering strategy to narrow down the most important and likely clinically relevant variants. For each variant, we collected annotations from databases including ClinVar and Exome Aggregation Consortium (ExAC, http://exac.broadinstitute.org)^36^. Following the ACMG Standards and Guidelines regarding classification and interpretation of sequence variants^37^, the variants were classified into five categories: Pathogenic Likely Pathogenic, Likely Benign, Benign and variants of unknown significance. Germline samples from the BCM-MDA cohort were previously analyzed^31^. None of the included BCM-MDA UTUC patients had a diagnosis of Lynch syndrome^31^.

### RNA extraction, RNAseq, and data analysis

RNA was extracted from frozen material for RNA-sequencing (RNA-seq) using Promega Maxwell 16 MDx instrument, (Maxwell 16 LEV simplyRNA Tissue Kit (cat. # AS1280)) from WCM UTUC tumors. Specimens were prepared for RNAseq using TruSeq RNA Library Preparation Kit v2 or riboZero. RNA integrity was verified using the Agilent Bioanalyzer 2100 (Agilent Technologies). cDNA was synthesized from total RNA using Superscript III (Invitrogen). Sequencing was then performed on GAII, HiSeq 2000, or HiSeq 2500. All reads were independently aligned with STAR_2.4.0f1^38^ for sequence alignment against the human genome sequence build hg19, downloaded via the UCSC genome browser (http://hgdownload.soe.ucsc.edu/goldenPath/hg19/bigZips/), and SAMTOOLS v0.1.19^39^ for sorting and indexing reads. Cufflinks (2.0.2)^40^ was used to estimate the expression values (FPKMS), and GENCODE v23^41^ GTF file for annotation. Rstudio (1.0.136) with R (v3.3.2) and ggplot2 (2.2.1) were used for the statistical analysis and the generation of figures. For fusion analysis, we used STAR-fusion (STAR-Fusion_v0.5.1)^42,43^. Fusions with significant support of junction reads and spanning pairs are then selected and manually reviewed. RNA was purified from BCM-MDA UTUC tumors and mRNA expression was computed for all genes from RNAseq data^31^. Gene fusions were detected in the RNAseq data using deFuse and SOAPfuse^31^.

### RNAseq data quantification, integration, and expression analysis

The mRNA gene expression for high-grade WCM UTUC and TCGA UCB tumors was quantified as Reads Per Kilobase of transcript per Million mapped reads (RPKMs). Similarly, RPKMs for high-grade tumors from a previously published UTUC cohort at BCM and MDACC were calculated^31^. The RPKMs from these three institutions were combined and quantile normalized to reduce any batch effects among the samples while maintaining their individual biological variability. The quantile normalized data were log transformed for further analyses. To rule out batch effects, we examined the normalized expression values for a set of 40 housekeeping genes expected to have comparable expression value distributions among the three datasets (WCM UTUC, BCM-MDA UTUC, TCGA UCB) (Supplementary Fig. 1). Differential gene expression (DGE) between UTUC and UCB cohorts was performed on the counts data using the Bioconductor package DESeq2. The threshold to select differentially regulated genes was determined at a fold change of >1 for upregulated and <−1 for downregulated genes and results were deemed significant at an adjusted *p*-value of 0.05 (Benjamini–Hochberg correction).

### Hierarchical clustering to infer UTUC subtypes

The subtypes in the UTUC cohorts, namely WCM and BCM-MDA, were inferred using previously published subtype-specific gene signatures together with previously reported^2^ subtype classifications for TCGA UCB samples. To this end, first, the normalized expression values corresponding to the BASE47-signature, MDACC-signature, and TCGA-signature genes were extracted from the three integrated data sets (namely WCM UTUC, BCM-MDA UTUC, and TCGA UCB). For each signature gene set, the subtype for each of the WCM UTUC and BCM-MDA UTUC samples was inferred as follows: (1) The samples were clustered based on Pearson correlation and average linkage, and the cophenetic distances among them were calculated, (2) TCGA samples with minimum cophenetic distances to each UTUC sample were identified, and corresponding TCGA subtype labels were assigned to the specific UTUC samples. Subsequently, the results corresponding to each signature gene set were visualized in heatmaps, scaled across signature genes, and grouped based on inferred subtypes.

### Unsupervised NMF

Unsupervised NMF was applied to the FPKM expression matrix for TCGA and UTUC samples. The input expression matrix was first filtered to retain only the top 25% of the genes with the highest expression variability. The optimal number of ranks was estimated to three based on 30 randomly initialized instances using the NMF R package. The NMF was then run with a rank *k* = 3 over 100 iterations, to obtain the final deconvolution into three resulting components. Amongst other genes, component 1 contained ECM/EMT markers (C7, COMP, DES, PGM5, SFRP4, CLDN3, TWIST1), component 2 contained luminal/papillary markers (CIS.Down (CRTAC1, CTSE), luminal (FGFR3, KRT20, SNX31, UPK1A, UPK2), and Sonic Hedgehog (SHH)), and component 3 contained basal/squamous markers (basal (KRT5, KRT14, KRT6A), immune (CXCL11, SAA1), and squamous (DSC3, GSDMC, PI3, TGM1). PCA was then performed on the coefficients obtained from NMF to visualize the separation of the three components.

### Outlier analysis

Genes expression outliers *Z*-scores were calculated for a list 74 cancer-related genes generated from the intersection of Sanger database and Drugbank (https://www.drugbank.ca/

https://www.sanger.ac.uk/science/tools/gdsc-genomics-drug-sensitivity-cancer). *Z*-scores were calculated across the WCM UTUC and BCM-MDA UTUC cohorts for these 74 cancer-related genes. For each sample, the quantiles were calculated and then used to compute the lower and upper bound to define an outlier. A cut-off of *Z-*score > 1 and FPKMS > 50 was reported for the UTUC outlier genes after comparison with TCGA UCB samples.

### Identification of T-cell-inflamed and T-cell-depleted subtypes

The top 5000 genes with the most variable normalized expression levels across TCGA UCB and UTUC samples (WCM and BCM-MDA) were selected based on their median absolute deviations. These genes data were then median centered and used as an input for hierarchal clustering and Euclidean distance (linkage = ward.D2, *k* = 20). A 170-gene cluster containing CD8A and other key immune genes was identified. A *k*-means consensus clustering of these 170 genes across both UTUC and UCB cohorts revealed the presence of two prominent subclusters that we labeled as T-cell inflamed (with higher expression of cluster genes) and T-cell depleted (with lower expression of cluster genes).

### Differential expression analysis of FGFR3 shRNA dataset

To study the role of FGFR3 in up-regulation of the interferon response, we obtained the publicly available Affymetrix microarray dataset from the RT-112 bladder cancer cell line, with or without shRNA-mediated knockdown of FGFR3^17,18^. The dataset comprised of 12 samples transduced with doxycycline-inducible shRNAs, which were either a shRNA-targeting EGFP (control) or one of three distinct shRNAs-targeting FGFR3 (FGFR3-shRNA); each condition had three biological replicates. This data was downloaded as raw signal intensity values for 54,675 probesets (Affymetrix Human Genome U133 Plus 2.0 Array). We used robust multiarray average (RMA) for background correction, normalization, and probe level intensity calculation from Affy Bioconductor Package (Version 1.52), in R statistical environment^44^. The normalized expression profiles were then used to identify differentially expressed probes between FGFR3-shRNA versus control samples using the limma package (version 3.30.13)^45^. Probes were collapsed to gene level after taking the median fold change of the probes, utilizing hgu133plus2 annotation data^46^. Genes that were differentially expressed after doxycycline induction in all three FGFR3-depleted cell lines but not in the control cell line were considered putative FGFR3-regulated genes. We identified 58 up-regulated genes (log-fold change > 1 and adjusted *P*-value < 0.05) and 45 downregulated genes. The log-fold change values of >1 or <−1 were used as thresholds to select for up-regulated or down-regulated genes, respectively (adjusted *P*-value < 0.05).

### Gene set enrichment analysis

A pre-ranked gene set enrichment analysis (GSEA) was applied to the differentially expressed genes, ordered based on their log-fold change values, to identify the cellular pathways significantly altered after shRNA-mediated knockdown of FGFR3^47^. Gene sets available through the Gene Ontology Biological Pathways collection in the Molecular Signatures Database^48^ were used for the GSEA analysis.

### Network analysis

Gene sets found to have a statistically significant enrichment using GSEA were visualized using network-based enrichment maps in Cytoscape^49^, where each node in the network was representative of an individual gene set^50^. In addition, the enrichment map also grouped redundant gene sets into distinct clusters enabling the identification of broader functional categories. The clusters from the enrichment maps were further refined and labeled using AutoAnnotate^51^. We only focused on cancer-related and immune-related pathways in the network.

### Real-time PCR

RT-4 and SW780 were purchased from ATCC (HTB-2 and CRL-2169) and RT-112 was purchased from Sigma (85061106). RT-4, SW780 and RT-112 were cultured in McCoy’s 5A (modified) medium (Thermo Fisher Scientific, 16600108), DMEM (Thermo Fisher Scientific, 11965118), and EMEM (ATCC, 302003) with 10% FBS, respectively. All the cell lines were mycoplasma negative and validated by STR testing. RT-112, RT4, and SW780 cells were treated with DMSO, Erdafitinib 1 μM, and Erdafitinib 5 μM for 48 hours, respectively. Total RNA was isolated with the RNeasy Plus Mini Kit (Qiagen #74134) according to the manufacturer’s protocol. Total RNA concentration was measured by NanoDrop (Thermo Fisher Scientific). cDNA was synthesized using SuperScript^TM^ III First-Strand Synthesis system (Invitrogen #18080051). Real-time PCR was performed by LightCycler 480 (Roche) using Power SYBR Green Master Mix (Applied Biosystems #4367659). All calculations were collected and analyzed with LightCycer 480 software (Roche) using the delta–delta Ct method. The reaction components, conditions, and primers used are listed in Supplementary Table 4. All data were normalized to the expression of the housekeeping gene beta-actin (*β-actin*) and then compared to the expression in the DMSO-treated group. Two independent experiments were performed, each with two technical replicates. The data were presented by mean ± SD. *P*-values were calculated using the *t*-test and corrected for multiple comparisons using the Holm–Sidak method. All analyses were performed using GraphPad Prism statistical software.

### Statistical analyses

For statistical tests, two-sided Mann–Whitney–Wilcoxon test was used to check for significant differences between two distributions. The two-sided Fisher’s exact test was applied to determine whether the deviations between the observed and the expected counts were significant. When appropriate, *P*-values were adjusted for multiple hypotheses testing with Benjamini–Hochberg procedure. Boxplot statistics were computed with the function “boxplot” of R programming language.

### Reporting summary

Further information on research design is available in the Nature Research Reporting Summary linked to this article.

## Supplementary information

Supplementary Information

Reporting Summary

Source Data

## Data Availability

The genomic data that support the findings of this study are available in the database of Genotypes and Phenotypes (dbGaP) and on cBioPortal for Cancer Genomics with the identifier https://www.cbioportal.org/study?id=utuc_cornell_baylor_mdacc_2019. The source data underlying Figs. 1a–d, 2a–e, 3a, b, and 4a–f and Supplementary Figs. 1, 2, 3 and 4 are provided as a Source Data file.
