# Supplementary material for: Upper tract urothelial carcinoma has a luminal-papillary T-cell depleted contexture and activated FGFR3 signaling
Source: Nat Commun. 2019 Jul 5;10:2977. doi: 10.1038/s41467-019-10873-y (PMC6611775; doi:10.1038/s41467-019-10873-y)
Supplement: Supplementary file 1 — Supplementary Information [file 41467_2019_10873_MOESM1_ESM.pdf]

Supplementary information to:

Upper tract urothelial carcinoma has a luminal-papillary T-cell depleted contexture and activated FGFR3 signaling.

by Robinson, Vlachostergios et al.

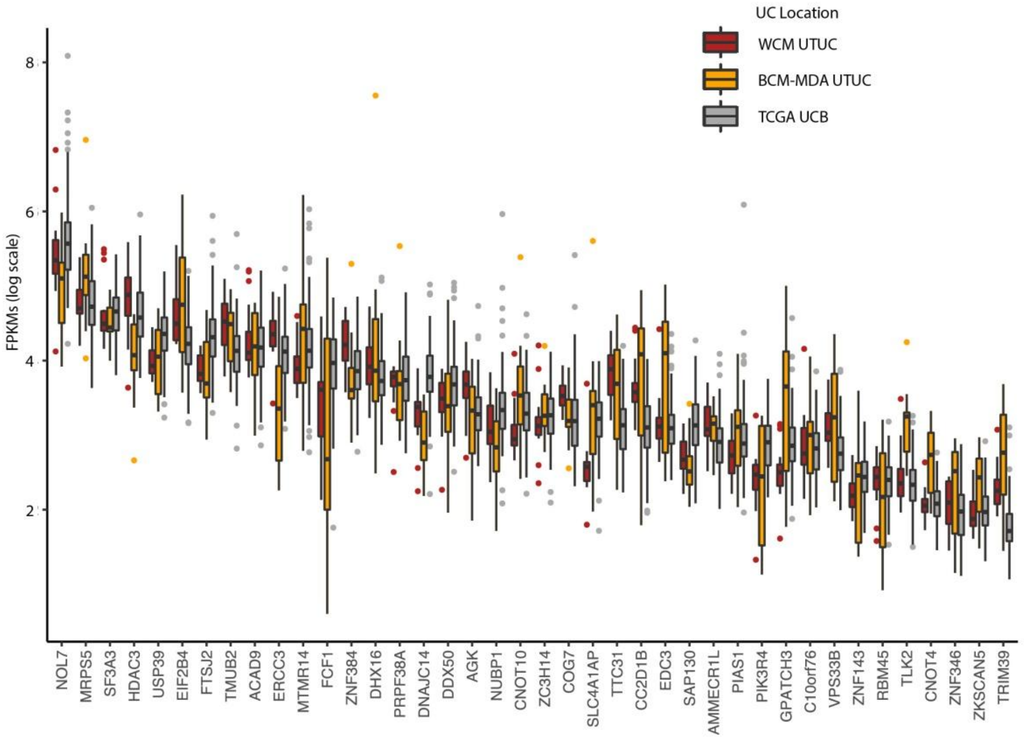

Supplementary Figure 1 Expression of housekeeping genes is similar across WCM UTUC, BCM-MDA UTUC, and TCGA UCB samples. Boxplot showing z-scores of the mRNA expression of 40 housekeeping genes among tumor samples from WCM UTUC (red bars), BCM-MDA UTUC (yellow bars) and TCGA UCB (gray bars) cohorts. The horizontal line within the box indicates the mean, boundaries of the box indicate the 25th- and 75th -percentile, and the whiskers indicate the highest and lowest values of the results.

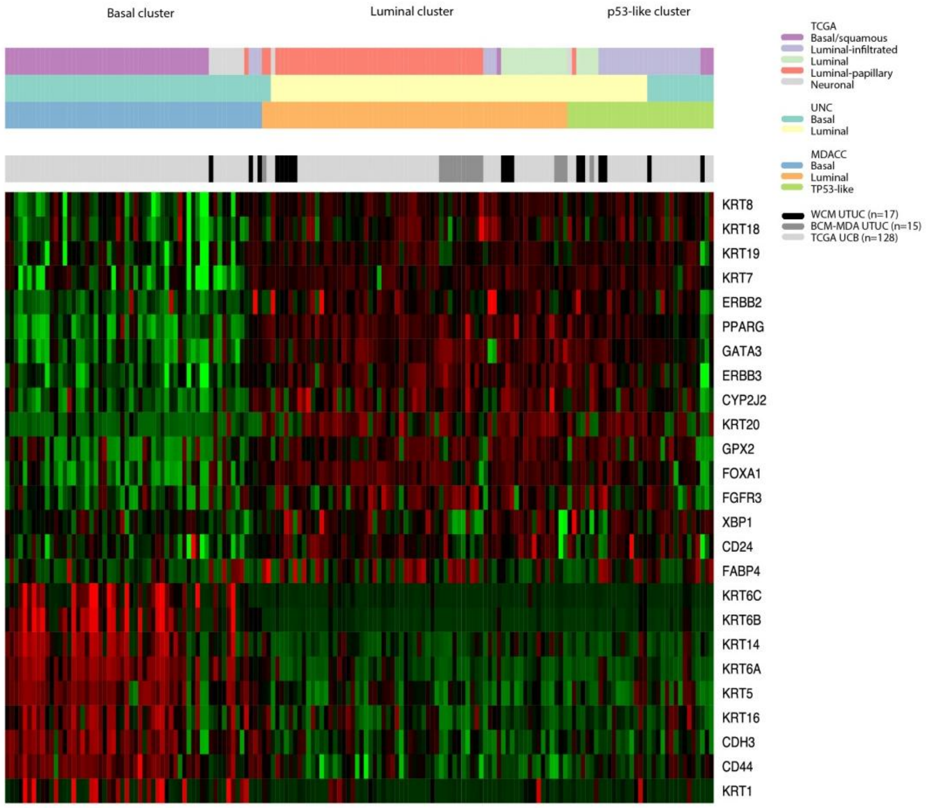

Supplementary Figure 2 UTUC is predominantly luminal by MDACC classification. Supervised consensus clustering and heatmap of mRNA expression data from WCM UTUC, BCM-MDA UTUC, and TCGA UCB metadataset. MDACC classifier genes are listed on the right. Assigned TCGA, MDACC, and UNC clusters are represented on top. WCM UTUC and BCM-MDA UTUC cluster with the luminal subtype (orange bars) by MDACC criteria, luminal subtype by UNC criteria (yellow bars) and luminal-papillary subtype by TCGA classification (red bars). Green = low, red = high mRNA expression.

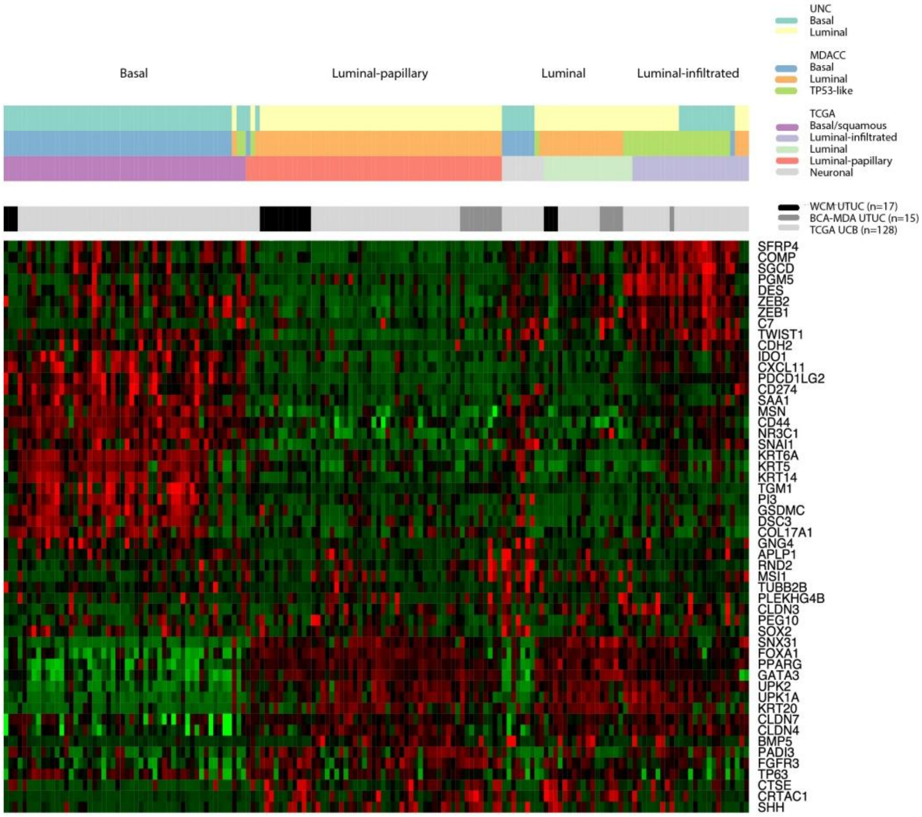

Supplementary Figure 3 UTUC is predominantly luminal-papillary by TCGA classification. Supervised consensus clustering and heatmap of mRNA expression from WCM UTUC, BCM-MDA UTUC, and TCGA UCB metadataset. TCGA classifier genes are listed on the right. Assigned TCGA, MDACC, and UNC clusters are represented on top. WCM UTUC and BCM-MDA UTUC cluster with the luminal-papillary subtype (red bars) by TCGA criteria, luminal subtype by UNC criteria (yellow bars) and luminal subtype by MDACC classification (orange bars). Green = low, red = high mRNA expression.

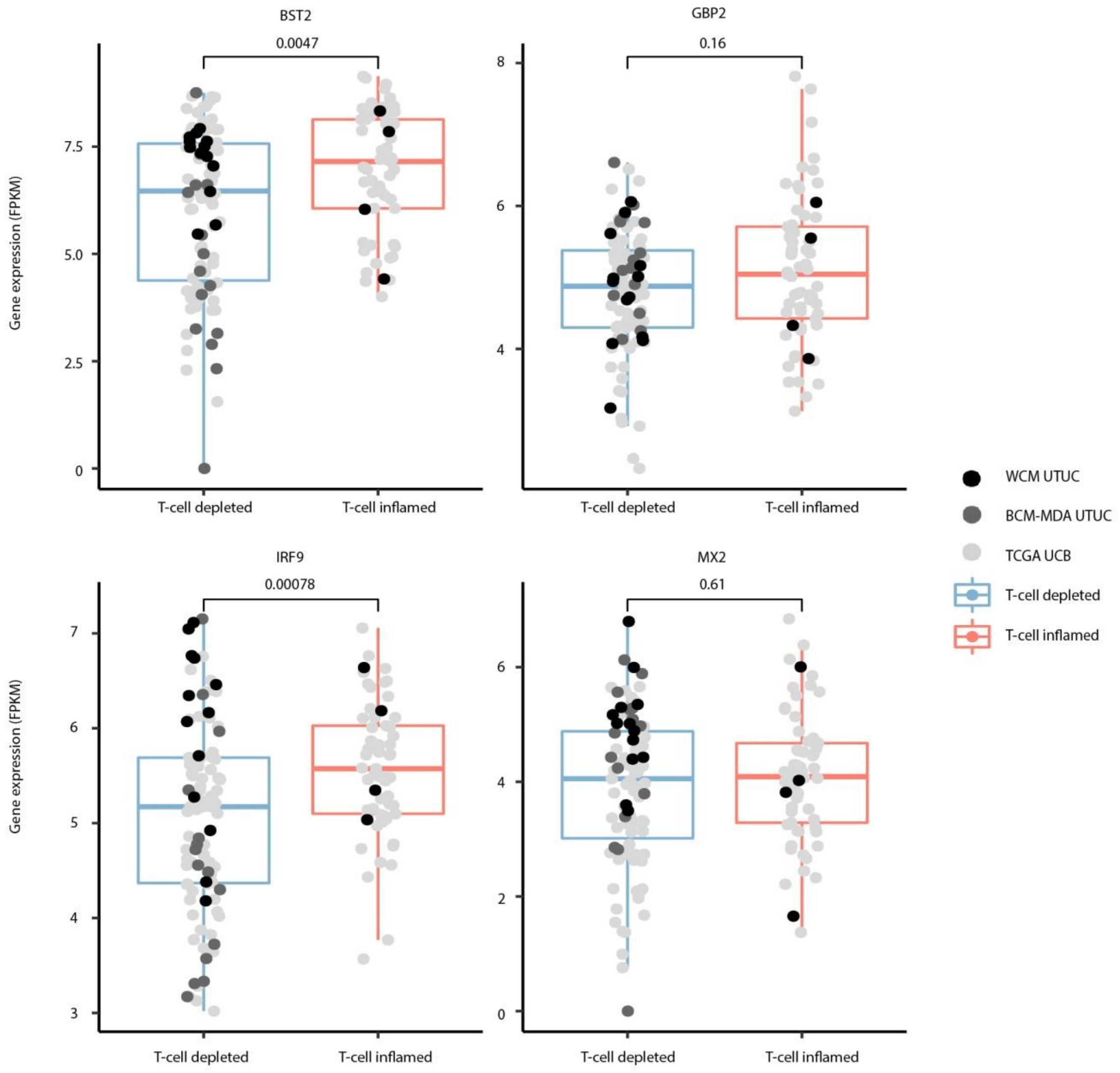

Supplementary Figure 4. *BST2* and *IRF9* are downregulated in T-cell UTUC tumors Boxplots of mean expression of *BST2*, *GBP2* (upper panel), *IRF9*, and *MX2* (lower panel) genes in FPKM within the T-cell depleted versus T-cell inflamed clusters of WCM UTUC, BCM-MDA UTUC, and TCGA UCB (BST2: Wilcoxon test $P = 0.0047$; IRF9 Wilcoxon test $P = 0.00078$). The horizontal line within the box indicates the mean, boundaries of the box indicate the 25th- and 75th -percentile, and the whiskers indicate the highest and lowest values of the results.

Supplementary Table 1.

Patient and sample characteristics.

UTUC ID	Age at diagnosis	Sex	Smoking status	Location	pT diagnosis <T2 vs ≥T2	Grade	History of bladder urothelial carcinoma	WES	RNAseq
WCM UTUC 21	85	F	Never	Renal pelvis	pT1	High	No	Yes	Yes
WCM UTUC 23	77	M	Former	Renal pelvis	pT4	High	No	Yes	No
WCM UTUC 25	55	F	Former	Renal pelvis	pT3	High	Yes	Yes	No
WCM UTUC 60	73	M	Former	Renal pelvis	pT1	High	No	Yes	No
WCM UTUC 70	57	F	Current	Renal pelvis	pT1	High	Yes	No	Yes
WCM UTUC 72	75	F	Current	Renal pelvis	pT3	High	Yes	Yes	No
WCM UTUC 73	82	M	Unknown	Renal pelvis	pT3	High	Yes	Yes	Yes
WCM UTUC 74	63	F	Never	Renal pelvis	pT3	High	No	No	Yes
WCM UTUC 75	58	F	Former	Renal pelvis	pT2	High	No	Yes	Yes
WCM UTUC 77	68	M	Never	Renal pelvis	pTa	High	Yes	Yes	Yes
WCM UTUC 78	66	M	Former	Ureter	pTa	High	Yes	No	Yes
WCM UTUC 80	71	M	Former	Renal pelvis	pT3	High	No	No	Yes
WCM UTUC 82	73	M	Former	Renal pelvis	pT3	High	Yes	No	Yes
WCM UTUC 83	87	F	Never	Ureter	pT3	High	Yes	No	Yes
WCM UTUC 84	85	M	Never	Renal pelvis	pT1	High	Yes	Yes	No
WCM UTUC 85	74	M	Former	Ureter	pT3	High	Yes	No	Yes
WCM UTUC 86	65	M	Never	Renal pelvis	pT3	High	No	No	Yes
WCM UTUC 87	70	M	Former	Renal pelvis	pT2	High	Yes	Yes	Yes
WCM UTUC 89	73	M	Former	Renal pelvis	pT1	High	Yes	Yes	Yes
WCM UTUC90	75	M	Former	Ureter	pT1	High	No	Yes	Yes
WCM UTUC 91	80	F	Never	Renal pelvis	pT3	High	No	Yes	Yes
WCM UTUC 92	84	F	Never	Ureter	pT4	High	No	Yes	Yes
WCM UTUC 576	74	M	Former	Ureter	pT3	High	Yes	Yes	No
WCM UTUC 761	51	M	Former	Ureter	pT1	High	Yes	Yes	No
WCM UTUC 764	73	M	Former	Ureter	pT3	High	Yes	Yes	No
WCM UTUC 784	64	M	Former	Renal pelvis	pT4	High	No	Yes	No
WCM UTUC 805	81	M	Former	Renal pelvis	pT3	High	Yes	Yes	No
WCM UTUC 885	81	M	Former	Ureter	pT3	High	No	Yes	No
WCMUTUC1099	73	F	Former	Renal pelvis	pT4	High	No	Yes	No
WCM UTUC 1399	82	M	Former	Renal pelvis	pT3	High	No	Yes	No
BCM-MDA 01 (IPCT-KC5-6867)	68	F	Never	Ureter	pT3	High	Yes	Yes	Yes
BCM-MDA 05 (IPCT-KC5-7975)	88	F	Never	Renal pelvis	pTa	High	No	Yes	Yes
BCM-MDA 09 (IPCT-KC5-7982)	83	M	Former	Renal Pelvis/Ureter	pT3	High	Yes	Yes	Yes
BCM-MDA 10 (IPCT-KC5-7983)	57	M	Never	Renal pelvis	pT1	High	No	Yes	Yes
BCM-MDA 12 (IPCT-KC5-7987)	58	F	Former	Renal pelvis	pT3	High	No	Yes	Yes
BCM-MDA 14 (IPCT-KC5-7990)	77	M	Never	Renal pelvis	cTa	High	No	Yes	Yes

BCM-MDA 16 (IPCT-KC5-7994)	76	M	Former	Renal pelvis	pT1	High	No	Yes	Yes
BCM-MDA 17 (IPCT-KC5-7996)	62	M	Former	Renal Pelvis/Ureter	pT2	High	No	Yes	Yes
BCM-MDA 18 (IPCT-KC5-7997)	76	M	Current	Renal pelvis	pT1	High	No	Yes	Yes
BCM-MDA 20 (IPCT-KC5-7999)	74	F	Never	Renal pelvis/Ureter	pT1	High	Yes	Yes	Yes
BCM-MDA 21 (UTUC-CAN-000203)	73	M	Current	Renal pelvis	pT1	High	Yes	Yes	Yes
BCM-MDA 22 (UTUC-CAN-000204)	78	M	Current	Renal pelvis	pT4	High	No	Yes	Yes
BCM-MDA 24 (UTUC-CAN-000207)	70	M	Current	Ureter	pT3	High	No	Yes	Yes
BCM-MDA 26 (UTUC-CAN-000223)	87	M	Current	Ureter	pT2	High	No	No	Yes
BCM-MDA 28 (UTUC-CAN-000225)	80	F	Current	Ureter	pT2	High	No	No	Yes
BCM-MDA 29 (IPCT-KC5-7988)	83	M	Never	Renal pelvis/Ureter	pT4	High	No	Yes	No
BCM-MDA 30 (IPCT-KC5-7995)	81	M	Never	Renal pelvis	pT3	High	No	Yes	No

Supplementary Table 2.

Inferred molecular subtypes per signature criteria, based on the UNC, MDACC, and TCGA classifier subtype of the sample with which highest Spearman correlation was found.

Type	SampleID	UNC	MDACC	TCGA
**UNC criteria**				
BCM-MDA	BCM-MDA 09	Basal	Basal	Basal squamous
BCM-MDA	BCM-MDA 24	Basal	TP53-like	Luminal infiltrated
WCM	WCM UTUC 74	Basal	Basal	Basal squamous
WCM	WCM UTUC 75	Basal	Basal	Basal squamous
WCM	WCM UTUC 92	Basal	Basal	Neuronal
BCM-MDA	BCM-MDA 01	Luminal	Luminal	Luminal
BCM-MDA	BCM-MDA 05	Luminal	Luminal	Luminal papillary
BCM-MDA	BCM-MDA 10	Luminal	Luminal	Luminal papillary
BCM-MDA	BCM-MDA 12	Luminal	Luminal	Luminal papillary
BCM-MDA	BCM-MDA 14	Luminal	Luminal	Luminal papillary
BCM-MDA	BCM-MDA 16	Luminal	Luminal	Luminal papillary
BCM-MDA	BCM-MDA 17	Luminal	Luminal	Luminal papillary
BCM-MDA	BCM-MDA 18	Luminal	Luminal	Luminal papillary
BCM-MDA	BCM-MDA 20	Luminal	Luminal	Luminal papillary
BCM-MDA	BCM-MDA 21	Luminal	Luminal	Luminal papillary
BCM-MDA	BCM-MDA 22	Luminal	Luminal	Luminal
BCM-MDA	BCM-MDA 26	Luminal	Luminal	Luminal papillary
BCM-MDA	BCM-MDA 28	Luminal	Luminal	Luminal
WCM	WCM UTUC 21	Luminal	TP53-like	Luminal papillary
WCM	WCM UTUC 70	Luminal	Luminal	Luminal papillary
WCM	WCM UTUC 73	Luminal	Luminal	Luminal papillary
WCM	WCM UTUC 77	Luminal	Luminal	Luminal papillary
WCM	WCM UTUC 78	Luminal	Luminal	Luminal papillary
WCM	WCM UTUC 80	Luminal	Luminal	Luminal papillary
WCM	WCM UTUC 82	Luminal	Luminal	Luminal papillary
WCM	WCM UTUC 83	Luminal	Luminal	Luminal papillary
WCM	WCM UTUC 85	Luminal	TP53-like	Luminal infiltrated
WCM	WCM UTUC 86	Luminal	Luminal	Luminal papillary
WCM	WCM UTUC 87	Luminal	Luminal	Luminal papillary
WCM	WCM UTUC 89	Luminal	Luminal	Luminal papillary
WCM	WCM UTUC 90	Luminal	Luminal	Luminal papillary
WCM	WCM UTUC 91	Luminal	Luminal	Luminal papillary
**MDACC criteria**				
WCM	WCM UTUC 74	Basal	Basal	Luminal infiltrated
WCM	WCM UTUC 75	Basal	Basal	Luminal infiltrated
WCM	WCM UTUC 80	Basal	Basal	Neuronal
BCM-MDA	BCM-MDA 24	Basal	Luminal	Luminal papillary

BCM-MDA	BCM-MDA 01	Luminal	Luminal	Luminal
BCM-MDA	BCM-MDA 17	Luminal	Luminal	Luminal
BCM-MDA	BCM-MDA 22	Luminal	Luminal	Luminal
WCM	WCM UTUC 21	Luminal	Luminal	Luminal
WCM	WCM UTUC 78	Luminal	Luminal	Luminal
WCM	WCM UTUC 83	Luminal	Luminal	Luminal
BCM-MDA	BCM-MDA 05	Luminal	Luminal	Luminal papillary
BCM-MDA	BCM-MDA 09	Luminal	Luminal	Luminal papillary
BCM-MDA	BCM-MDA 12	Luminal	Luminal	Luminal papillary
BCM-MDA	BCM-MDA 14	Luminal	Luminal	Luminal papillary
BCM-MDA	BCM-MDA 16	Luminal	Luminal	Luminal papillary
BCM-MDA	BCM-MDA 18	Luminal	Luminal	Luminal papillary
BCM-MDA	BCM-MDA 20	Luminal	Luminal	Luminal papillary
BCM-MDA	BCM-MDA 21	Luminal	Luminal	Luminal papillary
BCM-MDA	BCM-MDA 26	Luminal	Luminal	Luminal papillary
BCM-MDA	BCM-MDA 28	Luminal	Luminal	Luminal papillary
WCM	WCM UTUC 77	Luminal	Luminal	Luminal papillary
WCM	WCM UTUC 86	Luminal	Luminal	Luminal papillary
WCM	WCM UTUC 87	Luminal	Luminal	Luminal papillary
WCM	WCM UTUC 89	Luminal	Luminal	Luminal papillary
WCM	WCM UTUC 91	Luminal	Luminal	Luminal papillary
WCM	WCM UTUC 92	Basal	TP53-like	Basal squamous
WCM	WCM UTUC 85	Basal	TP53-like	Luminal infiltrated
BCM-MDA	BCM-MDA 10	Luminal	TP53-like	Luminal
WCM	WCM UTUC 70	Luminal	TP53-like	Luminal
WCM	WCM UTUC 82	Luminal	TP53-like	Luminal
WCM	WCM UTUC 73	Luminal	TP53-like	Luminal infiltrated
WCM	WCM UTUC 90	Luminal	TP53-like	Luminal infiltrated
**TCGA criteria**				
WCM	WCM UTUC 74	Basal	Basal	Basal squamous
WCM	WCM UTUC 75	Basal	Basal	Basal squamous
WCM	WCM UTUC 92	Basal	Basal	Basal squamous
BCM-MDA	BCM-MDA 14	Luminal	Luminal	Luminal
BCM-MDA	BCM-MDA 17	Luminal	Luminal	Luminal
BCM-MDA	BCM-MDA 21	Luminal	Luminal	Luminal
BCM-MDA	BCM-MDA 22	Luminal	Luminal	Luminal
BCM-MDA	BCM-MDA 28	Luminal	Luminal	Luminal
WCM	WCM UTUC 78	Luminal	Luminal	Luminal
WCM	WCM UTUC 83	Luminal	Luminal	Luminal
WCM	WCM UTUC 85	Luminal	Luminal	Luminal
BCM-MDA	BCM-MDA 24	Luminal	TP53-like	Luminal infiltrated
BCM-MDA	BCM-MDA 01	Luminal	Luminal	Luminal papillary
BCM-MDA	BCM-MDA 05	Luminal	Luminal	Luminal papillary
BCM-MDA	BCM-MDA 09	Luminal	Luminal	Luminal papillary
BCM-MDA	BCM-MDA 10	Luminal	Luminal	Luminal papillary

BCM-MDA	BCM-MDA 12	Luminal	Luminal	Luminal papillary
BCM-MDA	BCM-MDA 16	Luminal	Luminal	Luminal papillary
BCM-MDA	BCM-MDA 18	Luminal	Luminal	Luminal papillary
BCM-MDA	BCM-MDA 20	Luminal	Luminal	Luminal papillary
BCM-MDA	BCM-MDA 26	Luminal	Luminal	Luminal papillary
WCM	WCM UTUC 21	Luminal	Luminal	Luminal papillary
WCM	WCM UTUC 70	Luminal	Luminal	Luminal papillary
WCM	WCM UTUC 73	Luminal	Luminal	Luminal papillary
WCM	WCM UTUC 77	Luminal	Luminal	Luminal papillary
WCM	WCM UTUC 80	Luminal	Luminal	Luminal papillary
WCM	WCM UTUC 82	Luminal	Luminal	Luminal papillary
WCM	WCM UTUC 86	Luminal	Luminal	Luminal papillary
WCM	WCM UTUC 87	Luminal	Luminal	Luminal papillary
WCM	WCM UTUC 89	Luminal	Luminal	Luminal papillary
WCM	WCM UTUC 90	Luminal	Luminal	Luminal papillary
WCM	WCM UTUC 91	Luminal	Luminal	Luminal papillary

Supplementary Table 3.

Immune gene signature of 170 genes related to immune signaling that were differentially expressed between UTUC (WCM, BCM-MDA) and TCGA UCB.

MIR650	*FCGR3A*	*IDO1*	*ALOX5AP*	*SLC15A3*	*C3AR1*	*ELK2AP*	*HAVCR2*	*PILRA*	*EVI2A*
IGJ	*C1R*	*IFI30*	*CD3E*	*LTB*	*PTPRC*	*PLA2G7*	*RASSF4*	*IL4I1*	*CYTH4*
IGLL5	*HLA-DPA1*	*ITGB2*	*CPVL*	*HLA-DMA*	*ACP5*	*CD79A*	*NCKAP1L*	*GZMH*	*ADAP2*
CXCL10	*CD74*	*GZMB*	*GNLY*	*CD37*	*CD4*	*WIPF1*	*IL10RA*	*TRPV2*	*ABI3*
CXCL9	*HLA-DQB1*	*TYROBP*	*NKG7*	*CCL3*	*HCK*	*RNASE6*	*MSR1*	*CCR1*	*ITGAX*
LYZ	*SERPING1*	*IL2RG*	*CD2*	*TREM2*	*LCP2*	*CD48*	*F13A1*	*SAMSN1*	*RCSD1*
UBD	*HLA-DQA2*	*LAPTM5*	*GZMA*	*MS4A6A*	*CLEC7A*	*HCST*	*SASH3*	*IGSF6*	*TBXAS1*
C1S	*TMEM176A*	*C2*	*CD3D*	*CD7*	*EVI2B*	*CORO1A*	*SLAMF8*	*SLC7A7*	*LY86*
C1QB	*TMEM176B*	*CCL4*	*NCF2*	*FYB*	*SDS*	*SLAMF7*	*SLA*	*CD86*	*DOCK2*
CXCL13	*CCL5*	*SRGN*	*CYBB*	*SLCO2B1*	*HLA-DQB2*	*C1orf162*	*LST1*	*WAS*	*MYO1F*
C1QC	*HLA-DMB*	*CD52*	*VSIG4*	*WARS*	*FGL2*	*PRF1*	*GZMK*	*STAB1*	*PIK3AP1*
HLA-DRB1	*RGS1*	*FPR3*	*GBP5*	*MS4A4A*	*SELPLG*	*MNDA*	*BCL2A1*	*CD300A*	*PTPN7*
HLA-DRA	*FCGR2A*	*RNASE1*	*SPI1*	*AIF1*	*C5AR1*	*CCL13*	*CYTIP*	*SPOCK2*	*ITGAM*
HLA-DRB5	*CD14*	*CD53*	*CCL19*	*CST7*	*FAM26F*	*LAIR1*	*LAG3*	*CD8A*	*ADAMDEC1*
CCL21	*HLA-DRB6*	*MZB1*	*FOLR2*	*FPR1*	*HLA-DOA*	*IL2RB*	*CIITA*	*S100B*	*OSCAR*
C1QA	*FCER1G*	*CD163*	*IL7R*	*LY96*	*PLEK*	*IRF8*	*FERMT3*	*TNFSF13B*	*HK3*
HLA-DQA1	*HLA-DPB1*	*CXCL11*	*CSF1R*	*MAFB*	*GPR183*	*MPEG1*	*DOK2*	*ARHGAP9*	*LAT2*

Supplementary Table 4.

Primers, reaction components, and conditions for the quantitative real-time PCR in RT-112, RT-4, and SW780 cell lines.

**qPCR primers**		
Name	Sequence (5'-3')	
b-actin	F	TCCCTGGAGAAGAGCTACG
	R	GTAGTTTCGTGGATGCCACA
BST2 #1	F	CCGTCCTGCTCGGCTTT
	R	CCGCTCAGAACTGATGAGATCA
BST2 #2	F	ACGCGTCTGCAGAGGTGGAG
	R	GCAGCGGAGCTGGAGTCCT
**Reaction component (384 plate)**		
SYBR Green Mix (2X)	5	
Forward Primer (10uM)	0.25	
Reverse Primer (10uM)	0.25	
Template	50ng	
Nuclease-free water	To 10ul	
**qPCR condition (40 Cycles)**		
95C	10min	
95C	15sec	
55C	30sec	
60C	30sec	
