# Supplementary material for: Upper tract urothelial carcinoma has a luminal-papillary T-cell depleted contexture and activated FGFR3 signaling
Source: Nat Commun. 2019 Jul 5;10:2977. doi: 10.1038/s41467-019-10873-y (PMC6611775; doi:10.1038/s41467-019-10873-y)
Supplement: Supplementary file 2 — Reporting Summary [file 41467_2019_10873_MOESM2_ESM.pdf]

nature research

Corresponding author(s): Bishoy M. Faltas / NCOMMS-18-11881B

Last updated by author(s): Apr 18, 2019

Reporting Summary

Nature Research wishes to improve the reproducibility of the work that we publish. This form provides structure for consistency and transparency in reporting. For further information on Nature Research policies, see Authors & Referees and the Editorial Policy Checklist.

Statistics

For all statistical analyses, confirm that the following items are present in the figure legend, table legend, main text, or Methods section.

n/a	Confirmed	
☐	☒	The exact sample size (*n*) for each experimental group/condition, given as a discrete number and unit of measurement
☐	☒	A statement on whether measurements were taken from distinct samples or whether the same sample was measured repeatedly
☐	☒	The statistical test(s) used AND whether they are one- or two-sided<br>*Only common tests should be described solely by name; describe more complex techniques in the Methods section.*
☐	☒	A description of all covariates tested
☐	☒	A description of any assumptions or corrections, such as tests of normality and adjustment for multiple comparisons
☐	☒	A full description of the statistical parameters including central tendency (e.g. means) or other basic estimates (e.g. regression coefficient) AND variation (e.g. standard deviation) or associated estimates of uncertainty (e.g. confidence intervals)
☐	☒	For null hypothesis testing, the test statistic (e.g. *F*, *t*, *r*) with confidence intervals, effect sizes, degrees of freedom and *P* value noted<br>*Give P values as exact values whenever suitable.*
☐	☒	For Bayesian analysis, information on the choice of priors and Markov chain Monte Carlo settings
☐	☒	For hierarchical and complex designs, identification of the appropriate level for tests and full reporting of outcomes
☐	☒	Estimates of effect sizes (e.g. Cohen's *d*, Pearson's *r*), indicating how they were calculated

Our web collection on statistics for biologists contains articles on many of the points above.

Software and code

Policy information about availability of computer code

Data collection	Whole exome sequencing of WCM UTUC samples was performed using Illumina HiSeq 2500 (2×100 bp). A total of 21,522 genes were analyzed with an average coverage of 85× using Agilent HaloPlex Exome (Agilent Technologies, Santa Clara, CA). Bioinformatic analysis of BCM-MDA samples data was performed as previously described30. RNA sequencing of WCM UTUC samples was performed on GAII, HiSeq 2000, or HiSeq 2500. All reads were independently aligned with STAR_2.4.0f137 for sequence alignment against the human genome sequence build hg19, downloaded via the UCSC genome browser (http://hgdownload.soe.ucsc.edu/goldenPath/hg19/bigZips/), and SAMTOOLS v0.1.1938 for sorting and indexing reads. RNA was purified from BCM-MDA UTUC tumors and mRNA expression from was computed for all genes from RNA sequencing data.
Data analysis	All the WCM samples data were processed through the computational analysis pipeline of the Institute for Precision Medicine at Weill Cornell, New York Presbyterian Hospital (IPM-Exome-pipeline). Raw reads quality was assessed with FASTQC. Pipeline output includes segment DNA copy number data, somatic copy-number aberrations (CNAs) and putative somatic single nucleotide variants (SNVs). Bioinformatic analysis of BCM-MDA samples data was performed. For RNA sequencing analysis of WCM UTUC tumors, Cufflinks (2.0.2) was used to estimate the expression values (FPKMS), and GENCODE v2340 GTF file for annotation. Rstudio (1.0.136) with R (v3.3.2) and ggplot2 (2.2.1) were used for the statistical analysis and the generation of figures. mRNA expression from BCM-MDA UTUC tumors was computed for all genes from RNA sequencing data.

For manuscripts utilizing custom algorithms or software that are central to the research but not yet described in published literature, software must be made available to editors/reviewers. We strongly encourage code deposition in a community repository (e.g. GitHub). See the Nature Research guidelines for submitting code & software for further information.

Data

Policy information about availability of data

All manuscripts must include a data availability statement. This statement should provide the following information, where applicable:

- Accession codes, unique identifiers, or web links for publicly available datasets
- A list of figures that have associated raw data
- A description of any restrictions on data availability

The genomic data that support the findings of this study are available in the cBioPortal for Cancer Genomics with the identifier "https://www.cbioportal.org/study?id=utuc_cornell_baylor_mdacc_2019". The source data underlying Figs 1a-d, 2a–e, 3a,b and 4a-f and Supplementary Figs 1, 2, 3, and 4 are provided as a Source Data file.

Field-specific reporting

Please select the one below that is the best fit for your research. If you are not sure, read the appropriate sections before making your selection.

☒ Life sciences ☐ Behavioural & social sciences ☐ Ecological, evolutionary & environmental sciences

For a reference copy of the document with all sections, see nature.com/documents/nr-reporting-summary-flat.pdf

Life sciences study design

All studies must disclose on these points even when the disclosure is negative.

Sample size	47 tumor samples from 47 corresponding UTUC (WCM, BCM-MDA) patients were included in the study. WES analysis was performed in 37 samples from corresponding patients while RNAseq was performed in 32 samples from the total cohort.
Data exclusions	Patients with low-grade tumors, non-urothelial histology or variant histology in >50% of tumor tissue were excluded from the study.
Replication	Several methods were used for each analysis; for example for detection of UTUC subtypes 3 different classifiers and NMF were used, and results were further functionally studied in vitro.
Randomization	Not applicable to our study.
Blinding	Not applicable to our study.

Reporting for specific materials, systems and methods

We require information from authors about some types of materials, experimental systems and methods used in many studies. Here, indicate whether each material, system or method listed is relevant to your study. If you are not sure if a list item applies to your research, read the appropriate section before selecting a response.

Materials & experimental systems

n/a	Involved in the study	
☐	☒	Antibodies
☐	☒	Eukaryotic cell lines
☒	☐	Palaeontology
☒	☐	Animals and other organisms
☐	☒	Human research participants
☐	☒	Clinical data

Methods

n/a	Involved in the study	
☒	☐	ChIP-seq
☒	☐	Flow cytometry
☒	☐	MRI-based neuroimaging

Antibodies

Antibodies used	Mouse antibodies against MLH1 (G168-728, 1:25 dilution, BD Biosciences), PMS2 (A16-4, 1:100 dilution, BD Biosciences), MSH2 (FE11, 1:200, EMD Millipore), and MSH6 (44/MSH6, 1:200, BD Biosciences) were used.
Validation	G168-728 (citations):<br>1. Prolla TA, Christie DM, Liskay RM. Dual requirement in yeast DNA mismatch repair for MLH1 and PMS1, two homologs of the bacterial mutL gene. Mol Cell Biol. 1994; 14(1):407-415.<br>2. Prolla TA, Pang Q, Alani E, Kolodner RD, Liskay RM. MLH1, PMS1, and MSH2 interactions during the initiation of DNA mismatch repair in yeast. Science. 1994; 265(5175):1091-1093.<br><br>A16-4 (citations):<br>1. Cleaver JE. It was a very good year for DNA repair. Cell. 1994; 76(1):1-4.

2. Marsischky GT, Filosi N, Kane MF, Kolodner R. Redundancy of Saccharomyces cerevisiae MSH3 and MSH6 in MSH2-dependent mismatch repair. Genes Dev. 1996; 10(4):407-420.
3. Prolla TA, Christie DM, Liskay RM. Dual requirement in yeast DNA mismatch repair for MLH1 and PMS1, two homologs of the bacterial mutL gene. Mol Cell Biol. 1994; 14(1):407-415.
4. Prolla TA, Pang Q, Alani E, Kolodner RD, Liskay RM. MLH1, PMS1, and MSH2 interactions during the initiation of DNA mismatch repair in yeast. Science. 1994; 265(5175):1091-1093.
5. Su SS, Modrich P. Escherichia coli mutS-encoded protein binds to mismatched DNA base pairs. Proc Natl Acad Sci U S A. 1986; 83(14):5057-5061.

FE11 (citations):
1.Thibodeau SN, French AJ, Roche PC, Cunningham JM, Tester DJ, Lindor NM, Moslein G, Baker SM, Liskay RM, Burgart LJ, Honchel R, Halling KC.Altered expression of hMSH2 and hMLH1 in tumors with microsatellite instability and genetic alterations in mismatch repair genes. Cancer Res. 1996;56(21):4836-40.

44/MSH6 (citations):
1. Christmann M, Kaina B. Nuclear translocation of mismatch repair proteins MSH2 and MSH6 as a response of cells to alkylating agents. J Biol Chem. 2000; 275(46):36256-36262.
2. Humbert O, Hermine T, Hernandez H, et al. Implication of protein kinase C in the regulation of DNA mismatch repair protein expression and function. J Biol Chem. 2002; 277(20):18061-18068.
3. Kariola R, Otway R, Lonnqvist KE, et al. Two mismatch repair gene mutations found in a colon cancer patient--which one is pathogenic. Hum Genet. 2003; 112(2):105-109.
4. Palombo F, Gallinari P, Iaccarino I, et al. GTBP, a 160-kilodalton protein essential for mismatch-binding activity in human cells. Science. 1995; 268(5219):1912-1914.
5. Saitoh H, Pizzi MD, Wang J. Perturbation of SUMOlation enzyme Ubc9 by distinct domain within nucleoporin RanBP2/Nup358. J Biol Chem. 2002; 277(7):4755-4763.

Eukaryotic cell lines

Policy information about cell lines

Cell line source(s)	ATCC: RT-112, RT-4, SW780
Authentication	All cell lines tested were authenticated by STR testing, periodic morphology checks, growth curve analysis and testing for Mycoplasma contamination.
Mycoplasma contamination	All cell lines tested were negative for Mycoplasma contamination.
Commonly misidentified lines (See ICLAC register)	All cell lines tested were authenticated by STR testing.

Human research participants

Policy information about studies involving human research participants

Population characteristics	Patient demographics and clinical characteristics are described in Supplementary Table 1.
Recruitment	Fresh frozen and formalin fixed paraffin embedded (FFPE) samples were retrospectively collected from banked excess tissue from nephroureterectomy archival specimens of patients with a diagnosis of high-grade upper tract urothelial carcinoma (UTUC). UTUC high-grade samples were obtained from patients under protocols approved by institutional review boards using endoscopic biopsy or surgical resection at Baylor College of Medicine (BCM) and MD Anderson Cancer Center (MDA), as previously described30. All tumor samples consisted of conventional UC. Samples were selected based on pathologic diagnosis according to standard guidelines for UTUC1. All pathology specimens were reviewed and reported by board-certified genitourinary pathologists in the Department of Pathology at WCM/NYP, BCM and MDA. Clinical charts were reviewed by the authors to record patient demographics, tobacco use, treatment history, anatomic site, the presence of concurrent bladder cancer, pathologic grade and stage using tumor, node, metastasis (TNM) system.
Ethics oversight	Weill Cornell Medicine (WCM) / New York-Presbyterian (NYP), Baylor College of Medicine (BCM) and MD Anderson Cancer Center (MDA).

Note that full information on the approval of the study protocol must also be provided in the manuscript.

Clinical data

Policy information about clinical studies

All manuscripts should comply with the ICMJE guidelines for publication of clinical research and a completed CONSORT checklist must be included with all submissions.

Clinical trial registration	Not applicable in our study.
Study protocol	Weill Cornell Medicine (WCM) / New York-Presbyterian (NYP) IRB protocols for Tumor Biobanking – 0201005295, GU tumor Biobanking – 1008011210, Urothelial Cancer Sequencing – 1011011386, and Precision Medicine - 1305013903). Protocols approved by institutional review boards using endoscopic biopsy or surgical resection at Baylor College of Medicine (BCM) and MD Anderson Cancer Center (MDA).

Data collection	Data were retrospectively collected between 2012 and 2018 across 3 different institutions (WCM, BCM-MDA).
Outcomes	Not applicable in our study.
